# Evaluation of Thymol-β-d-Glucopyranoside as a Potential Prebiotic Intervention to Reduce Carriage of Zoonotic Pathogens in Weaned and Feeder Pigs

**DOI:** 10.3390/microorganisms9040860

**Published:** 2021-04-16

**Authors:** Gizem Levent, Robin C. Anderson, Branko Petrujkić, Toni L. Poole, Haiqi He, Kenneth J. Genovese, Michael E. Hume, Ross C. Beier, Roger B. Harvey, David J. Nisbet

**Affiliations:** 1 United States Department of Agriculture, Agricultural Research Service, Food and Feed Safety Research Unit, College Station, TX 77845, USA; GLevent@cvm.tamu.edu (G.L.); Petrujkic@vet.bg.ac.rs (B.P.); Toni.Poole@usda.gov (T.L.P.); Haiqi.He@usda.gov (H.H.); Kenneth.Genovese@usda.gov (K.J.G.); Michael.Hume@usda.gov (M.E.H.); Ross.Beier@usda.gov (R.C.B.); Roger.Harvey@usda.gov (R.B.H.); David.Nisbet@usda.gov (D.J.N.); 2 Department of Veterinary Pathobiology, Texas A&M University, College Station, TX 77840, USA; 3 Faculty of Veterinary Medicine, University of Belgrade, 11000 Belgrade, Serbia

**Keywords:** *Campylobacter*, *Escherichia coli*, *Salmonella*, swine, thymol, thymol-β-d-glucopyranoside

## Abstract

The gut of food-producing animals is a reservoir for foodborne pathogens. Thymol is bactericidal against foodborne pathogens but rapid absorption of thymol from the proximal gut precludes the delivery of effective concentrations to the lower gut where pathogens mainly colonize. Thymol-β-d-glucopyranoside is reported to be more resistant to absorption than thymol in everted jejunal segments and could potentially function as a prebiotic by resisting degradation and absorption in the proximal gut but being hydrolysable by microbial β-glycosidase in the distal gut. Previous in vitro studies showed bactericidal effects of thymol-β-d-glucopyranoside against *Campylobacter*, *Escherichia coli*, and *Salmonella enterica* serovar Typhimurium in the presence but not absence of intestinal microbes expressing β-glycosidase activity, indicating that hydrolysis was required to obtain antimicrobial activity. Presently, the oral administration of thymol-β-d-glucopyranoside was studied to examine the effects on intestinal carriage of *Campylobacter*, *E. coli*, and *S.* Typhimurium in swine. The effects of thymol-β-d-glucopyranoside or thymol on antimicrobial sensitivity of representative *E. coli* isolates and characterized *Salmonella* strains were also explored. Results from two in vivo studies revealed little antimicrobial effects of thymol-β-d-glucopyranoside on *Campylobacter*, *E. coli*, or *S.* Typhimurium in swine gut. These findings add credence to current thinking that hydrolysis and absorption of thymol-β-d-glucopyranoside and thymol may be sufficiently rapid within the proximal gut to preclude delivery to the distal gut. Antibiotic susceptibilities of selected bacterial isolates and strains were mainly unaffected by thymol. Further research is warranted to overcome obstacles, preventing the delivery of efficacious amounts of thymol-β-d-glucopyranoside to the lower gut.

## 1. Introduction

*Campylobacter* and non-typhoidal *Salmonella* are leading bacterial causes of human foodborne illness that are estimated to cause 1.5 and 1.35 million human infections, respectively, every year in the United States [1,2]. Poultry, swine, and cattle can harbor these pathogens within their digestive tracts and risk contaminating carcasses during processing. In recent years, the increasing number of antibiotic-resistant *Campylobacter* and *Salmonella* isolates recovered from human infections has also become a serious public health threat [1]. Antibiotic use in these animals may select for antibiotic resistant bacteria which can result in serious public health consequences [3]. Therefore, a non-antibiotic pre-harvest strategy is needed to reduce the risk of carcass contamination at the abattoir and also to reduce the risk of selection of antibiotic resistant pathogens that can pose a greater risk for public health.

Essential oils such as thymol are considered attractive alternatives to antibiotics due to their bactericidal activities against a variety of pathogens [4]. Thymol is a component of the essential oil of thyme that exerts its bactericidal activity via insertion into the lipid-rich bacterial cell wall and subsequent disintegration of microbial cell wall integrity [5,6,7]. Thymol has been shown to be effective against zoonotic pathogens such as *Salmonella*, *Campylobacter*, as well as against many enterotoxigenic *Escherichia coli*, such as those responsible for disease in young pigs [8], with minimum inhibitory concentrations against pure cultures ranging from 1.00 and 1.55 µmol/mL [5,7,9]. In addition to its bactericidal activity, thymol has been also shown to increase the sensitivity of pathogens against antibiotics thus acting as a potential solution to combat antibiotic resistance [10,11]. Conversely, essential oil extracts from *Dittrichia graveolens* and rich in oxygenated monoterpenes have been reported to be antagonistic against the antimicrobial activity of streptomycin [12]. Results from in vivo studies, however, have shown marginal antimicrobial activity of free thymol against foodborne pathogens in the gastrointestinal tract of swine due potentially to its rapid absorption or degradation in the stomach and small intestine of monogastric animals [13,14,15]. A more stable, conjugated form of thymol, thymol-β-d-glucopyranoside, has recently been investigated as a potential prebiotic to promote the passage of the thymol derivative to the cecum and large intestine [16,17,18,19]. Conceptually, it was hypothesized that because of its β-glycosidic bond and larger size thymol-β-d-glucopyranoside would be resistant to hydrolysis and absorption in the stomach and the proximal small intestine of monogastric animals yet readily hydrolysable by microbial β-glycosidases, abundantly expressed by autochthonous bacteria in the distal gut [20,21]. Evidence in support of the hypothesis that thymol-β-d-glucopyranoside is more resistant to absorption than free thymol was reported by Petrujkić et al. [19] where approximately 2.5 times more free thymol was absorbed by everted porcine jejunal segments than thymol-β-d-glucopyranoside. Additionally, Epps et al. [17] reported that microbial β-glycosidase activity expressed by a common gut bacterium, *Parabacteroides distasonis*, rapidly hydrolyzed thymol-β-d-glucopyranoside to yield free thymol which was then able to exert potent anti-*Campylobacter* activity during co-culture with *C. jejuni* and *C. coli*. When cultured alone, and thus in the absence of β-glycosidase activity, intact thymol-β-d-glucopyranoside exerted no antimicrobial activity against *C. jejuni* and *C. coli*.

Epps and colleagues [16] also compared the effects of oral administration of thymol or thymol-β-d-glucopyranoside (each to achieve approximately 1 mM of luminal concentration) on viable counts of indigenous *Campylobacter* and *E. coli* in the crop and ceca of market-aged broilers. They recovered 1.1 to 1.5 log_10_ fewer *Campylobacter*/g crop content from broilers treated with thymol-β-d-glucopyranoside compared to thymol-treated or untreated broilers but observed no effect of treatment on the recovery of *Campylobacter* from the ceca. The authors suggested rapid hydrolysis of thymol-β-d-glucopyranoside by crop microbiota may have concurrently hastened the absorption of the liberated thymol and depleted effective concentrations of the thymol conjugate thereby preventing efficient passage through the stomach and small intestine [16]. Significant effects of thymol or thymol-β-d-glucopyranoside were not observed on the recovery of *E. coli* from either crop or cecal contents collected from the broilers, which the authors attributed as potentially being due to a dose too low for this bacterial population [16]. Results from in vitro studies, for instance, revealed that a dose of 1 mM thymol-β-d-glucopyranoside was effective against *Campylobacter* [16,17] but doses of 3 and 6 mM thymol-β-d-glucopyranoside were needed to achieve equivalent activity against *Salmonella* Typhimurium and *E. coli* K88 [18] when cultured with intestinal microbiota recovered from chickens or pigs. As predicted, thymol-β-d-glucopyranoside at such doses exhibited little to no antimicrobial activity against these pathogens when cultured in pure culture due to the absence of appreciable β-glycosidase activity. Following up on this research, the objectives of the present study were to explore potential in vivo effects of multiple concentrations of 6 mM thymol-β-d-glucopyranoside on experimentally inoculated populations of *S.* Typhimurium population and indigenous *E. coli* populations in the swine gastrointestinal tract. In addition, to investigate potential effects of thymol on antibiotic susceptibility, selected wildtype *E. coli* isolates recovered from the pigs and three characterized *Salmonella* strains were tested against a panel of antibiotics before and after potential exposure to thymol-β-d-glucopyranoside or the hydrolyzed aglycone, thymol.

## 2. Materials and Methods

### 2.1. Animals, Challenge Salmonella, and Thymol-β-d-Glucopyranoside

For in vivo studies, all pigs (male and female progeny of Landrace x Yorkshire dams crossed with Pietrain x Duroc x Hampshire sires) were obtained from a local producer and upon delivery to our facility, were cared for according to a protocol approved by the Southern Plains Agricultural Research Center’s Institutional Animal Care and Use Committee (ACUC Protocol #2104005 approved October 15, 2014). All pigs were found to be culture-negative for the presence of wildtype *Salmonella* and all pigs in the first study were found to be culture-negative for *Campylobacter* via qualitative culture of rectal swabs (2/pig) collected upon their arrival to our rearing facility. Qualitative culture for *Salmonella* was accomplished via pre-enrichment of one of the swabs collected from each pig in tetrathionate broth (Difco, Becton Dickinson, Sparks, MD, USA), secondary enrichment in Rappaport-Vassiliadis broth (Difco), and selective differentiation on novobiocin-supplemented (25 µg/mL) Brilliant Green Agar (Oxoid, Unipath Ltd., Basinstoke, Hampshire, England) as previously described [22]. Cultivation of the remaining rectal swab of each pig for wildtype *Campylobacter* was accomplished by streaking to Campy-Cefex agar prepared and used as described by Stern et al. [23]. Since we had anticipated these pigs to be culture-negative for wildtype *Salmonella*, a *S.* Typhimurium (NVSL 95-1776), possessing natural resistance to novobiocin (Nov), and having been made nalidixic acid resistant (Nal) via successive cultivation in tryptic soy broth containing up to 20 µg of nalidixic acid/mL [24], was used as an experimental challenge strain in our studies. Inocula for experiments were obtained from *S.* TyphimuriumNovr-Nalr cultures grown 24 h at 37 °C in tryptic soy broth (Difco) supplemented with 25 µg of novobiocin/mL and 20 µg of nalidixic acid/mL. Novobiocin and nalidixic acid were purchased from Sigma-Aldrich (St. Louis, MO, USA); thymol-β-d-glucopyranoside was purchased from Christof Senn Laboratories, Dielsdorf, Switzerland.

### 2.2. In Vivo Study Designs

In the first pig study, 18 weaned pigs (24 ± 5 kg live body weight) were orally gavaged with 2 × 10^9^ colony forming units (CFU) of the *S.* TyphimuriumNovr-Nalr approximately 2 h after arrival (11:00) to the rearing facility and randomly allocated to 6 pens (3 pigs/pen) and twice-treated via oral gavage (2 pens/treatment) that same day (16:00 and 21:00) with 0, 6, or 18 mg of thymol-β-d-glucopyranoside/kg body weight. Pigs were provided ad libitum access to water and a non-medicated starter diet prepared by Luedemann Feed & Farm, (Brenham, TX, USA) composed (weight %) of 65% ground corn, 14.1% soybean meal, 4.8% lysine, 0.1% Mycolock NC-500 (Trouw Nutrition USA, Strykersville, NY, USA), and 0.1% selenium. This was the same diet used by the farm that provided the pigs. Pigs were euthanized 12 h after the last treatment and cecal and rectal contents collected at necropsy were cultured to enumerate the *S.* TyphimuriumNovr-Nalr and wildtype *E. coli* and *Campylobacter*.

In a second pig study, 12, 11, and 12 weaned pigs, respectively, averaging 40 ± 8 kg live body weight, were randomly allocated to a single day’s feed treatment of 0, 18, or 52 mg thymol-β-d-glucopyranoside/kg live body weight. All, except 1, of the pigs were placed (2 per pen) to concrete-floored pens, with the remaining pig placed individually in a similar pen. Pigs were acclimated to water and the commercially-prepared non-medicated starter diet provided ad libitum via twice-a-day feedings (08:00 and 16:00) for 14 days. On the evening of the 14th day, all pigs were orally inoculated via gavage with approximately 2 × 10^9^ CFU of the *S.* TyphimuriumNovr-Nalr. Treatments were administered via two feedings (08:00 and 16:00) to each pen on the 15th day via the top dressing half of the day’s treatment into a 20% portion of each meal, which was offered first to achieve total consumption of the thymol-β-d-glucopyranoside. Approximately 30 min later the remainder of the meal was offered. Pigs not receiving thymol-β-d-glucopyranoside were fed each meal in two portions likewise. Sixteen h and again 24 h after administration of the last meal, one pig from each pen was euthanized and necropsied for collection of cecal and rectal contents so that *n* = 6, 6, and 6 pigs for 0, 18, and 54 mg/kg live body weight per day at 16 h post treatment and *n* = 6, 5, and 6 for 0, 18, and 54 mg/kg live body weight per day at 24 h post treatment, respectively. Gut contents were cultured to enumerate the *S.* TyphimuriumNovr-Nalr and wildtype *E. coli* and *Campylobacter*.

### 2.3. Bacterial Enumerations

Gut samples collected from pigs at necropsy were serially diluted (10-fold) in 0.1-M sodium phosphate buffer (pH 6.5) and plated on Brilliant Green agar supplemented with 25 µg of novobiocin/mL and 20 µg of nalidixic acid/mL and on MacConkey (Difco) agar for viable cell count of the *S.* TyphimuriumNovr-Nalr and generic *E. coli*, respectively. Samples collected in the second pig study were also plated to Campy Cefex agar for enumeration of generic *Campylobacter* species. *Salmonella* TyphimuriumNovr-Nalr and *E. coli* were counted after 24 h of aerobic incubation at 37 °C; *Campylobacter* were enumerated after 48 h of microaerophilic (N_2_:CO_2_:O_2_; 85:10:5) incubation at 42 °C.

### 2.4. Phenotypic Antibiotic Susceptibility

An assessment of potential co-selection of increased or decreased antibiotic susceptibility by thymol, the hydrolytic product of thymol-β-d-glucopyranoside, was performed using randomly chosen strains of presumptive wildtype *E. coli* isolated from rectal swabs of each treatment group of the second in vivo study (18 and 54 mg of thymol-β-d-glucopyranoside/kg body weight). Rectal swabs were collected from 6 pigs of each treatment group before treatment administration (pre-exposure isolates) and from the same pigs (3 pigs/treatment) after 16 h and again after 24 h of treatment (3 pigs/treatment) administration (16 h-post exposure and 24 h-post exposure isolates, respectively). The presumptive pre-treatment *E. coli* were recovered following 24 h of incubation of MacConkey plates streaked with rectal swabs collected immediately before the first feed administration treatments and presumptive post-treatment *E. coli* were recovered from rectal swabs collected immediately before the euthanasia of the pigs necropsied at 16 and 24 h post treatment.

Additionally, multi-drug resistant *S. enterica* strains characterized as serovars Give (isolate 24349) and Typhimurium (isolates 22544 and 20731) and graciously provided by Dr. Shaohua Zhao (Office of Research, Center for Veterinary Medicine, Food, and Drug Administration, Laurel, MD. USA) [25] were used to assess potential effects of thymol exposure on antimicrobial sensitivity. These strains, having no known prior exposure to thymol, were chosen to avoid potentially confounding effects associated with the *S.* Typhimurium Novr-Nalr challenge strain which was prominent in post-treatment rectal populations. Phenotypic antibiotic susceptibility of these strains was previously reported by Zhao et al. [25]. To provide sublethal exposure of these *Salmonella* strains, they were adapted via three consecutive-24 h cultures in Thermo Scientific Sensititre^TM^ Cation Adjusted Mueller-Hinton broth w/TES (Remel Inc., Lenexa, KS, USA) supplemented with 0.5 mM thymol. Isolates before and after thymol exposure were assessed for their phenotypic antibiotic susceptibility.

Antibiotic susceptibility testing of isolates were performed according to CLSI standards [26] against 18 antibiotics (ampicillin, ceftiofur, chlortetracycline, clindamycin, danofloxacin, enrofloxacin, florfenicol, gentamicin, neomycin, oxytetracycline, penicillin, spectinomycin, sulphadimethoxime, tiamulin, tilmicosin, trimethoprim/sulphamethoxazole, tulathromycin, and tylosin tartrate) using the broth microdilution method and Sensititre Bovine/Porcine (BOPO6F) plates and Sensititre system (Trek Diagnostics System, UK). Susceptibilities are reported as minimum inhibitory concentrations (MIC) to allow for comparisons in the absence of antimicrobial resistance breakpoints, which for the antibiotics tested are not available for porcine gastrointestinal isolates reported here. Antimicrobial resistance breakpoints (S = sensitive, I = intermediate, and R = resistant) corresponding to ampicillin, ceftiofur, gentamicin, and trimethoprim/sulphamethoxazole are available for humans and are (MIC) S < 8, I = 16, R > 32; S < 2, I = 4, R > 8; S < 4, I = 8, R > 16, S < 2/38, I = not available, and R > 4/76, respectively [27].

### 2.5. Statistical Analysis

In the first animal study, control and thymol-β-d-glucopyranoside treatments were administered independently via oral gavage to each pig. Accordingly, log_10_ transformations of viable counts (CFU/g gut contents) of *S.* TyphimuriumNovr-Nalr and generic *E. coli* were analyzed for the effects of treatment using an analysis of variance. Polynomial contrasts were used to examine linear and quadratic effects of the treatment level. In the second study, control and thymol-β-d-glucopyranoside treatments were administered communally to pigs within each pen randomly allocated to receive its respective treatment and then one pig from each pen was collected for necropsy at 16 h post treatment with the remaining pig in each pen being collected for necropsy 24 h post treatment. Consequently, each pen served as the experimental unit and viable counts (log_10_ CFU/g) of *S.* TyphimuriumNovr-Nalr, generic *E. coli*, and *Campylobacter* species were analyzed for the main effects of treatment and time using a repeated measures analysis of variance and least significant difference (LSD) separation of means. All statistical analyses were completed with Statistix10 Analytical Software (Tallahassee, FL, USA). Due the availability of too few isolates for antimicrobial susceptibility testing, pre-exposure and post-exposure comparisons of MIC values are presented descriptively without statistical analysis.

## 3. Results and Discussion

### 3.1. In Vivo Pig Studies

Results from two separate animal studies revealed that oral administration of thymol-β-d-glucopyranoside had little if any effect on concentrations of the *Salmonella* TyphimuriumNovr-Nalr orally inoculated into the pigs and likewise had little if any effect on gut concentrations of generic *E. coli* or *Campylobacter* species (Table 1 and Table 2). In the first study, for instance, cecal but not rectal concentrations of the *S.* TyphimuriumNovr-Nalr were reduced in a linear (*p* < 0.05), dose-dependent fashion by treatment (Table 1). Neither cecal nor rectal concentrations of wildtype *E. coli* were affected by treatment and *Campylobacter* were not recovered from any of the pigs of the first study. The absence of a treatment effect on cecal *E. coli* populations while there was an observed treatment effect on *S.* TyphimuriumNovr-Nalr likely reflects the lower sensitivity of *E. coli* to the bactericidal effect of free thymol. These doses were calculated to deliver, based on an estimated 200 mL of intestinal volume, a total dose of approximately 2.3 to 6.9 µmol of thymol-β-d-glucopyranoside/g content to the lumen of the pig gut if no absorption were to have occurred. In our second study, which was done to assess the potential impact of an older distal gut flora on the antimicrobial activity of thymol-β-d-glucopyranoside, we examined the use of two dose levels (similarly calculated, based on an estimated 400 mL of intestinal volume, to deliver approximately 5.4 to 16.3 µmol of thymol-β-d-glucopyranoside/g to the lumen of the pig gut). Additionally, these doses were administered at two intervals to allow the broadening of sampling intervals. Despite the administration of doses equivalent to or approximately 3-fold higher than the dosed used in the first study and longer intervals between the administration of the last dose and sample collection at necropsy, evidence of an efficacious treatment effect was not observed. A treatment effect was observed on rectal *E. coli* populations but in this case rectal contents from pigs treated with 18 mg of thymol-β-d-glucopyranoside/g body weight had higher *E. coli* populations than pigs not treated or treated with 54 mg of thymol-β-d-glucopyranoside/g body weight (Table 2).

The lack of an efficacious effect of thymol-β-d-glucopyranoside suggests that adequate amounts of thymol-β-d-glucopyranoside or free thymol were not delivered to the cecum or large intestine. Possible reasons for this could be that hydrolysis of thymol-β-d-glucopyranoside and the subsequent absorption of free thymol may have been sufficiently rapid within the proximal small intestine to preclude the delivery of the intact glucopyranoside to the cecum and large intestine. Results from earlier in vitro incubations indicated that there was indeed appreciable thymol-β-d-glucopyranoside hydrolytic activity, as evidenced by anti-*Salmonella* activity, in populations of swine jejunal microbes [28]. Similarly, it is also possible that despite being >2-times more resistant to absorption than free thymol in everted jejunal segments [19], appreciable quantities of thymol-β-d-glucopyranoside administered in the present study may still have been absorbed in the small intestine. Van Noten and colleagues [29], in work published subsequent to the completion of our live animal studies, reported that appreciable amounts of thymol-α-d-glucopyranoside or thymol-β-d-glucopyranoside were hydrolyzed by microbial activity in the proximal gut of pigs. The thymol-conjugates in their study were fed at doses of approximately 42 mg/kg body weight administered over six meals each administered 2 h apart to pigs similar in weight to those used in our first in vivo study, Van Noten et al. [29] further suggested that neither glucopyranoside was completely resistant from absorption in the stomach or to further hydrolysis and absorption in the small intestine, with only traces, if any, of the glucopyranosides or free thymol being recovered in the small intestine or more distal portions of the intestinal tract [29]. Based on their analysis, Van Noten et al. [29] concluded that in the absence of absorption the maximal amounts of thymol and thymol-α-d-glucopyranoside expected to reach the small intestine would be approximately 0.3 µmol/g digesta. Their results further indicated that thymol-β-d-glucopyranoside may be absorbed more rapidly in the stomach than thymol-α-d-glucopyranoside thus indicating even lesser amounts of thymol-β-d-glucopyranoside would pass to the small intestine. Results from our study add credence to their suggestion that rapid hydrolysis of thymol-β-d-glucopyranoside likely precludes the delivery of the conjugate to the lower gut. For instance, based on the results of Van Noten et al. [29] it is likely that appreciable amounts of the 18 or 54 mg of thymol-β-d-glucopyranoside/body weight administered in the feed in our second study would be hydrolyzed. Subsequent absorption of the liberated thymol thus would have precluded the delivery of thymol-β-d-glucopyranoside at or above the 1, 3, and 6 µmol/g of gut content, respectively, reported to achieve effective antimicrobial activity against *Campylobacter*, *Salmonella*, and *E. coli* in vitro [16,17,18]. Contrarily, our observed effect of 18 mg of thymol-β-d-glucopyranoside/kg body weight on cecal *S.* TyphimuriumNovr-Nalr in our first study, achieving a 1.75 log_10_-fold decrease in viable counts, may reflect differences due to the method of administrations used in the two studies. For instance, experimental administration of treatments via oral gavage of a liquid-based treatment, while not practical in production settings, likely promoted more rapid passage of the intact glucopyranoside through the stomach and small intestine than feed administration. Subsequent retention within the cecum would be expected to persist longer than in the stomach and small intestine and hydrolysis of thymol-β-d-glucopyranoside by gut microbes therein would liberate free thymol, thereby allowing opportunity to act on *Salmonella* residing in this site.

It is possible, however, that uptake and internal compartmentalization of thymol-β-d-glucopyranoside by gut bacteria, or the lipophilic properties of both thymol and, albeit to a lesser extent, thymol-β-d-glucopyranoside, may sequester these compounds away from hydrolytic enzymes thus preventing the release of free thymol. Evidence for the former mechanism has been reported by Beier et al. [30] who found that rapid bacterial uptake of thymol-β-d-glucopyranoside can occur. In the case of the latter mechanism, results from in vitro studies, for instance, indicated that lipids and fatty acids inhibited the bactericidal activity of thymol-β-d-glucopyranoside in mixed populations of gut bacteria, thus implicating a potential role of the lipophilicity of both thymol and thymol-β-d-glucopyranoside [31].

Considering that mean retention times of digesta passage through the pig stomach, small intestine and total tract are estimated to be 1, 4, and more than 30 h, respectively [32], it may be possible to feed higher oral doses of thymol-β-d-glucopyranoside than those used here or by Van Noten et al. [29] to avoid complete hydrolysis of the administered doses in the stomach and small intestine yet persist long enough to reach the distal gut to exert antimicrobial activity against zoonotic pathogens upon activation by microbial thymol-β-d-glucopyranoside-hydrolyzing enzymes. Alternatively, it may be possible to use glucopyranoside compounds synthesized with disaccharides or other larger molecular weight carbohydrate moieties that may be more resistant to hydrolysis and absorption in the proximal gut. Additional research is currently underway to try and overcome these possible obstacles.

### 3.2. Antimicrobial Susceptibilities

Results from tests assessing the potential for thymol exposure to co-select for presumptive *E. coli* isolated from the pigs before and after being fed 18 or 54 mg of thymol-β-d-glucopyranoside/kg body weight exhibiting increased or decreased susceptibility to antibiotics included on Sensititre Bovine/Porcine (BOPO6F) plates are presented in Table 3, Table 4 and Table 5. The results provided limited evidence that prior exposure to thymol-β-d-glucopyranoside, and potentially its hydrolysis product thymol, may co-select for increased or decreased susceptibility to the antibiotics tested. In most cases, changes in antibiotic resistance profiles were not observed between the presumptive *E. coli* isolates having had no prior exposure or having been previously exposed to thymol-β-d-glucopyranoside.

We were able to detect the potential for at least 4-fold or greater increases in MIC values corresponding to ampicillin and ceftiofur in presumptive *E. coli* recovered from pigs fed 18 mg of thymol-β-D-glucopyranoside/kg body weight (post-exposure) when compared to presumptive *E. coli* recovered from the same pigs before exposure to thymol-β-D-glucopyranoside (pre-exposure) (Table 3).

Similarly, in comparisons between presumptive *E. coli* isolates recovered from pigs after or before being fed 52 mg of thymol-β-d-glucopyranoside/kg body weight (post- and pre-exposure, respectively), we detected the potential for at least 4-fold or greater increases in MIC values to ampicillin, ceftiofur, as well as to danofloxacin, enrofloxacin, spectinomycin, and tulathromycin. Conversely, we observed decreased susceptibility of at least 4-fold for oxytetracycline, neomycin, tulathromycin, and spectinomycin in post-exposed presumptive *E. coli* isolates compared to their corresponding pre-exposed isolates (Table 4). In a number of instances, results for increased or decreased susceptibility were undiscernible due to at least one MIC value being unrestrictedly greater or lesser than the pre- or post-exposure comparison (Table 3 and Table 4).

Of the three multi-drug resistant *Salmonella* strains tested before or after repeated exposure to sublethal amounts of thymol, which is the active agent of thymol-β-d-glucopyranoside, we observed no differences in MIC values pre- or post-thymol exposure (Table 5). It is important, however, to recognize that increases of 2- to 4-fold are not necessarily remarkable as these may well be within the variability of the study method and thus may not reflect consequential effects of thymol-β-d-glucopyranoside or its hydrolysis product thymol.

## 4. Conclusions

The gut of pigs can be colonized with important foodborne and disease-causing bacteria such as *Campylobacter*, *E. coli*, and *Salmonella*. New treatments and strategies are sought to reduce the carriage of these bacteria particularly as they are shipped to the processing plant. Thymol is an attractive candidate to be developed into an antibiotic alternative for swine because it is a natural product and thus likely to be viewed favorably by regulatory agencies. Thymol is known to exhibit potent antimicrobial activity against *Campylobacter*, *E. coli*, and *Salmonella* in the laboratory but its effectiveness when fed to animals is modest and inconsistent. This is because thymol is very rapidly absorbed in the proximal gastrointestinal tract, which consequently prevents it from arriving to the cecum and large intestine where these pathogenic bacteria primarily reside. In the present report, we tested a conjugated form of thymol, thymol-β-d-glucopyranoside, for its potential bypass absorption in the stomach and small intestine and make its way to the cecum and large intestine. Results from live animal studies revealed a modest (1.75 log_10_-fold decrease) decrease in viable counts of an experimentally inoculated *S.* Typhimurium in weaned pigs orally gavaged with 18 mg of thymol-β-d-glucopyranoside/kg body weight but no decrease in wildtype *E. coli* counts. Feed administration of up to 54 mg thymol-β-d-glucopyranoside/kg body weight was not efficacious in decreasing counts of wildtype *Campylobacter*, *E. coli*, or the challenge *S.* Typhimurium strain. The lack of efficacy is likely because hydrolysis and absorption of thymol-β-d-glucopyranoside and its hydrolysis product, free thymol, may still have been rapid enough within the proximal small intestine to preclude their delivery to the cecum and large intestine. A comparison of antimicrobial resistance profiles between presumptive *E. coli* isolates or multidrug resistant *Salmonella* strains provided no evidence that exposure to thymol-β-d-glucopyranoside or thymol co-selected for strains were more or less susceptible to antibiotics. Additional research is currently underway to try and learn how to overcome obstacles preventing the efficacious activity of thymol-β-d-glucopyranoside to the lower gastrointestinal tract.

## Figures and Tables

**Table 1:** Effect of oral thymol-β-d-glucopyranoside treatment on gut novobiocin- and nalidixic acid-resistant Salmonella Typhimurium (*S.* TyphimuriumNovr-Nalr) and generic *E. coli* in weaned swine.

	Thymol-β-d-Glucopyranoside Treatment (mg/kg Live Body Weight)			*p* Values		
	None	6	18	Linear	Quadratic	SEM
*S.* TyphimuriumNovr-Nalr (log_10_ CFU/g gut contents) ^a^						
Cecal	3.67 ^a^	3.26 ^a^	1.92 ^b^	0.0272	0.8004	0.528
Rectal	3.50	2.98	2.84	0.4671	0.6896	0.578
*Escherichia coli*^b^ (log_10_ CFU/g gut contents) ^a^						
Cecal	6.89	6.86	7.17	0.3976	0.6879	0.252
Rectal	6.72	7.10	7.25	0.1913	0.5381	0.257

^a^*n* = 6 pigs/treatment were twice-treated via oral gavage on the same day (16:00 and 21:00) with 0, 6, or 12 mg of thymol-β-d-glucopyranoside/kg body weight and sampled 12 h after the last treatment for bacteriological cultivation of cecal and rectal contents collected via necropsy to enumerate the *S.* TyphimuriumNovr-Nalr and generic *E. coli*. Means did not differ based on least significant difference (LSD) separation of means (*p* > 0.05). ^a,b^ Means within row with unlike superscript letters differ based on a LSD separation of means (*p* < 0.05).

**Table 2:** Effect of oral thymol-β-d-glucopyranoside treatment on gut novobiocin- and nalidixic acid-resistant *Salmonella* Typhimurium (*S.* TyphimuriumNovr-Nalr) and generic *E. coli* and *Campylobacter* species in weaned swine.

	Thymol-β-d-Glucopyranoside Treatment ^a^								
	Treatment Level (mg/kg Live Body Weight)		Hours Post Treatment			Main Effect (*p* Values)			
	None	18	54	16	24	Treatment	Time	Interaction	SEM
*S.* Typhimurium_Novr-Nalr_ (log_10_ CFU/g) ^a^									
Cecal	2.70	2.77	2.64	2.20 ^e^	3.21 ^d^	0.9599	0.0291	0.8778	0.539
Rectal	2.10	2.10	1.37	2.00	1.72	0.2692	0.4537	0.7558	0.482
*Escherichia coli* (log_10_ CFU/g)									
Cecal	5.29	5.88	5.37	5.26	5.77	0.2300	0.2479	0.8596	0.549
Rectal	4.73 ^b,c^	5.54 ^b^	4.35 ^c^	5.01	4.73	0.0100	0.3833	0.6126	0.398
*Campylobacter* species (log_10_ CFU/g)									
Cecal	4.04	4.13	3.52	3.67	4.13	0.2139	0.1235	0.2652	0.368
Rectal	3.48	3.76	3.57	3.23 ^e^	3.98 ^d^	0.8090	0.0150	0.3263	0.352

^a^*n* = 6, 6, and 6 pigs were fed a one-day administration of 0, 18, and 54 mg of thymol-β-d-glucopyranoside/kg live body weight per day via two equal feedings (08:00 and 16:00). Gut samples were collected 16 and 24 h after the last administration for bacteriological cultivation of cecal and rectal contents collected via necropsy to enumerate the *S.* TyphimuriumNovr-Nalr and wildtype *E. coli* and *Campylobacter* species, ^b,c^ Treatment means within row with unlike superscript letters differ based on a LSD separation of means (*p* < 0.05), ^d,e^ Time of sampling means within row with unlike superscript letters differ based on a LSD separation of means (*p* < 0.05).

**Table 3:** Antimicrobial susceptibilities of presumptive *E. coli* isolated from rectal swabs of pigs before or after feed administration of 18 mg of thymol-β-d-glucopyranoside/kg body weight.

	Presumptive *E. coli* Isolates Recovered Before (Pre-) and 16 h After (Post-) Treatment						Presumptive *E. coli* Isolates Recovered Before (Pre-) and 24 h After (Post-) Treatment				
	Pen 1		Pen 3		Pen 14		Pen 1	Pen 3		Pen 14	
Antibiotic ^a^	Pre- Exposure	Post- Exposure	Pre- Exposure	Post- Exposure	Pre- Exposure	Pre- Exposure	Post- Exposure	Pre- Exposure	Post- Exposure	Pre- Exposure	Post- Exposure
AMP	4	2	1	2	4	4	4	1	4 ^b^	2	2
XNL	≤0.25	≤0.25	≤0.25	1 ^b^	0.5	0.5	0.5	≤0.25	0.5 ^c^	≤0.25	0.5 ^c^
CTET	>8	>8	>8	>8	>8	>8	>8	>8	>8	>8	>8
DANO	≤0.12	≤0.12	≤0.12	≤0.12	≤0.12	≤0.12	≤0.12	≤0.12	≤0.12	≤0.12	≤0.12
ENRO	≤0.12	≤0.12	≤0.12	≤0.12	≤0.12	≤0.12	≤0.12	≤0.12	≤0.12	≤0.12	≤0.12
FFN	4	8	8	8	4	4	4	8	4	4	8
NEO	≤4	≤4	≤4	≤4	≤4	≤4	≤4	≤4	≤4	≤4	≤4
OXY	>8	>8	>8	>8	>8	>8	>8	>8	>8	>8	> 8
PEN	>8	>8	>8	>8	>8	>8	>8	8	>8	>8	>8
SPE	32	>64	16	>64	16	16	16	16	16	16	16
SDM	≤256	>256 ^c^	>256	>256	≤256	≤256	>256 ^c^	>256	≤256 ^c^	<256	≤256 ^c^
TIL	64	64	64	64	64	>64	64	32	64	64	64
TUL	4	4	4	8	8	8	4	2	4	4	4

^a^ AMP, ampicillin; XNL, ceftiofur; CTET, chlortetracycline; DANO, danofloxacin; ENRO, enrofloxacin; FFN, florfenicol; NEO, neomycin; OXY, oxytetracycline; PEN, penicillin; SPE, spectinomycin; SDM, sulfadimethoxine; TIL, tilmicosin; and TUL, tulathromycin. MIC values for clindamycin; gentamicin; tiamulin, trimethoprim/sulfamethoxazolem and tylosin tartrate were for all isolates > 16, ≤ 1, > 32, ≤ 2, and > 32, respectively. ^b^ Presumptive *E. coli* isolates recovered from same pen with post-thymol-β-d-glucopyranoside exposure MIC values at least 4-fold greater than the corresponding pre-thymol-β-d-glucopyranoside exposure MIC values. ^c^ Undiscernable comparison result due to at least one MIC value being unrestrictedly lesser or greater than the corresponding value.

**Table 4:** Antimicrobial susceptibilities of presumptive *E. coli* isolated from rectal swabs of pigs before or after feed administration of 54 mg of thymol-β-d-glucopyranoside/kg body weight.

		Presumptive *E. coli* Isolates Recovered Before (Pre-) and 16 h After (Post-) Treatment					Presumptive *E. coli* Isolates Recovered Before (Pre-) and 24 h After (Post-) Treatment					
		Pen 5	Pen 8		Pen 10		Pen 5		Pen 8		Pen 10	
Antibiotic ^a^	Pre- Exposure	Post- Exposure	Pre- Exposure	Post- Exposure	Pre- Exposure	Post- Exposure	Pre- Exposure	Post- Exposure	Pre- Exposure	Post- Exposure	Pre- Exposure	Post- Exposure
AMP	4	4	4	>16 ^b^	4	2	4	2	2	1	2	8 ^b^
XNL	0.5	0.5	0.5	≤0.25 ^c^	0.5	≤0.25 ^c^	0.5	≤0.25 ^c^	0.5	1	≤0.25	8 ^b^
CTET	>8	>8	>8	>8	>8	>8	>8	>8	>8	4	>8	4
DANO	≤0.12	≤0.12	≤0.12	≤0.12	≤0.12	≤0.12	≤0.12	≤0.12	≤0.12	0.5 ^b^	≤0.12	≤0.12
ENRO	≤0.12	≤0.12	≤0.12	≤0.12	≤0.12	≤0.12	≤0.12	≤0.12	≤0.12	0.5 ^b^	≤0.12	≤0.12
FFN	4	4	4	4	4	8	4	4	8	4	8	4
NEO	≤4	≤4	≤4	≤4	32	≤4 ^b^	≤4	≤4	≤4	≤4	≤4	≤4
OXY	>8	>8	>8	>8	>8	>8	>8	>8	>8	2 ^b^	>8	2 ^b^
PEN	>8	>8	>8	>8	>8	>8	>8	>8	>8	8	>8	>8
SPE	16	16	16	16	16	>64 ^b^	16	16	>64	64	>64	16 ^b^
SDM	≤256	>256 ^c^	≤256	≤256	>256	>256	>256	≤256	>256	>256	>256	≤256 ^c^
TIL	64	64	64	64	>64	64	64	64	64	>64	64	>64 ^c^
TUL	4	4	4	4	16	4^d^	4	4	8	8	4	16 ^b^

^a^ AMP, ampicillin; XNL, ceftiofur; CTET, chlortetracycline; DANO, danofloxacin; ENRO, enrofloxacin; FFN, florfenicol; NEO, neomycin; OXY, oxytetracycline; PEN, penicillin; SPE, spectinomycin; SDM, sulfadimethoxine; TIL, tilmicosin; TUL, tulathromycin. MIC values for clindamycin; gentamicin; tiamulin, trimethoprim/sulfamethoxazolem, and tylosin tartrate were for all isolates > 16, ≤ 1, > 32, ≤ 2, and > 32, respectively. ^b^ Presumptive *E. coli* isolates recovered from same pen with post-thymol-β-d-glucopyranoside exposure MIC values at least 4-fold greater or lesser than the corresponding pre-thymol-β-d-glucopyranoside exposure MIC values. ^c^ Undiscernable comparison due to at least one MIC value being unrestrictedly lesser or greater than the corresponding value.

**Table 5:** Antimicrobial resistance profiles of stock multidrug resistant *Salmonella enterica* isolates provided by the US-FDA before and after intentional exposure to 0.5 mM of thymol.

	*Salmonella enterica* Serovar Before (Pre-) and After (Post-) Repeated Exposure to 0.5 mM of Thymol					
	Pre-Exposure	Post-Exposure	Pre-Exposure	Post-Exposure	Pre-Exposure	Post-Exposure
Antibiotic ^a^	Give 24349	Give 24349	Typhimurium 22544	Typhimurium 22544	Typhimurium 20731	Typhimurium 20731
AMP	1	1	>16	>16	>16	>16
XNL	0.5	0.5	1	0.5	>8	>8
CTET	>8	>8	>8	>8	>8	>8
DANO	≤0.12	≤0.12	≤0.12	≤0.12	≤0.12	≤0.12
ENRO	≤0.12	≤0.12	≤0.12	≤0.12	≤0.12	≤0.12
FFN	2	2	4	4	8	8
NEO	≤4	≤4	≤4	≤4	32	32
OXY	>8	>8	>8	>8	>8	>8
PEN	8	8	>8	>8	>8	>8
SPE	32	32	32	32	>64	>64
SDM	>256	>256	>256	>256	>256	>256
TIL	>64	>64	>64	>64	>64	>64
SXT	≤2	≤2	≤2	>2 ^b^	≤2	≤2
TUL	4	8	64	64	16	16

^a^ AMP, ampicillin; XNL, ceftiofur; CTET, chlortetracycline; DANO, danofloxacin; ENRO, enrofloxacin; FFN, florfenicol; NEO, neomycin; OXY, oxytetracycline; PEN, penicillin; SPE, spectinomycin; SDM, sulfadimethoxine; TIL, tilmicosin; SXT, trimethoprim/sulfamethoxazole; and TUL, tulathromycin. MIC values for clindamycin; gentamicin; tiamulin and tylosin tartrate were for all isolates > 16, ≤ 1, > 32, and > 32, respectively. ^b^ Undiscernable comparison result due to at least one MIC value being unrestrictedly lesser or greater than the corresponding value.

## Data Availability

Not applicable.
